# C-reactive Protein as an Indicator for Antidepressant Response in Late-Onset Depression

**DOI:** 10.7759/cureus.90133

**Published:** 2025-08-15

**Authors:** Umesh Pratap Singh, Dheerendra Kumar Mishra, Ujwal Sardesai

**Affiliations:** 1 Medicine, Shyam Shah Medical College, Rewa, IND; 2 Psychiatry, Government Medical College Satna, Satna, IND; 3 Psychiatry, Mahatma Gandhi Memorial Medical College, Indore, IND

**Keywords:** antidepressant, c-reactive protein, late-life depression, late-onset depression, vascular depression

## Abstract

Background

Late-onset depression is pathophysiologically distinct from early-onset depression, often requiring higher doses and a longer duration of antidepressant therapy to achieve a therapeutic response. Immune dysfunction, atherosclerosis, and vascular etiology are critical factors involved in the pathogenesis of late-onset depression. Based on this understanding, we hypothesize that levels of inflammatory markers in individuals with late-onset depression may be associated with their response to antidepressant therapy.

Methodology

Individuals aged >60 years who presented with their first depressive episode (as defined by the International Classification of Diseases, Tenth Revision, Diagnostic Criteria for Research) were recruited. A complete clinical assessment, C-reactive protein (CRP) level, and depression severity assessment using the Hamilton Depression Rating Scale (HAMD-17) were performed at baseline. Patients were prescribed antidepressant medication and reassessed for depression severity in HAMD after an eight-week follow-up.

Results

The study sample (n = 25) had a mean age of 64.7 ± 5.8 years and a baseline HAMD score of 18 ± 3. The overall response rate to antidepressant therapy was 24%. The mean age of individuals who responded to antidepressant therapy (n = 6) was 63.5 ± 4.9 years, and their baseline HAMD score was 16 ± 1.9. The mean age of individuals who were partial responders or non-responders to antidepressant therapy (n = 19) was 65.1 ± 6.1 years, and their baseline HAMD score was 18.5 ± 3.9. Additionally, there was a negative correlation between baseline CRP levels and antidepressant responsiveness (r = -0.6, p < 0.05).

Conclusions

Late-onset depression was less responsive to antidepressant medication, and a poor antidepressant response rate was associated with a higher level of CRP in late-onset depression.

## Introduction

Depression significantly contributes to the global burden of disease, impacting quality of life, productivity, and physical health. Over 264 million people worldwide are affected, making it a leading cause of disability [1]. Late-onset depression (LOD), also known as geriatric depression, emerges in individuals aged 60 years or older. It is the second most common psychiatric disorder among the elderly, with a prevalence estimated at 10% to 20% [2,3]. LOD differs from early-onset depression (EOD) in etiology, presentation, treatment, and outcome [4]. LOD is associated with increased morbidity; suicide risk; impaired physical, cognitive, and social functioning; and higher self-neglect, all contributing to increased mortality [5]. Depression has a highly recurrent long-term course, resulting in substantial costs for patients, caregivers, and society [6].

There are various treatment modalities available for managing a depressive episode, including antidepressant medications, somatic therapies, and psychotherapeutic interventions. However, pharmacotherapy is the most commonly used treatment modality for depression worldwide [7].

Although there have been significant developments in the understanding of the psychopharmacology and biomarkers of major depression and the introduction of new classes of antidepressants, the effectiveness of these treatments remains limited [8]. In fact, over 80% of patients either do not respond adequately to therapy or experience early relapse within the first 6-12 weeks. The Sequenced Treatment Alternatives to Relieve Depression (STAR*D) study found that 50%-66% of patients with depression do not achieve full recovery with antidepressant medication, and only one-third of patients experience a remission of their depressive symptoms [9].

Antidepressant treatment in LOD differs from EOD because individuals with LOD are more susceptible to the side effects of antidepressant medication [10]. Moreover, they often experience greater difficulty in tolerating therapeutic doses and may require a longer duration of treatment to respond to the medication when compared to those with EOD [11]. Research has shown that LOD is associated with a poorer prognosis than depression in younger patients, with almost 40% of individuals with LOD experiencing chronic or continuously recurrent depression [12].

As of now, no biological marker has been identified or studied to evaluate antidepressant response in LOD. Treatment response is typically defined as a significant reduction in Hamilton Rating Scale for Depression (HAMD-17) score, usually at least 50% [13]. LOD, also known as vascular depression, is closely associated with vascular risk factors and elevated inflammatory mediators such as C-reactive protein (CRP) [14]. Elevated CRP levels are correlated with the severity of depressive episodes, particularly in women. Twin studies suggest a common genetic pathway linking depression and inflammation [15]. Variants of the CRP gene can influence circulating CRP levels and act as independent risk factors for LOD [16]. CRP levels have been used for monitoring various diseases for decades, and with the development of highly sensitive tests, it has become possible to measure levels of this protein within the normal range [17]. Based on this, it is hypothesized that the use of CRP may be useful in predicting antidepressant responses in individuals with LOD.

## Materials and methods

Study design and setting

This investigation was conducted as a prospective, longitudinal cohort study over a six-month period (July 2016 to December 2016) in the outpatient Department of Psychiatry at MGM Medical College, Indore (Madhya Pradesh), India. Elderly patients (>60 years) presenting with clinical features suggestive of depression were enrolled consecutively after a comprehensive psychiatric evaluation and were followed for eight weeks to evaluate antidepressant response and its association with baseline CRP levels.

Subject recruitment and eligibility

The study included individuals aged 60 years and above who were diagnosed with LOD, defined as the first onset of a major depressive episode after the age of 60 years, as per the criteria laid down in the International Classification of Diseases, 10th Revision (ICD-10). All patients were either treatment-naïve or had not received antidepressant therapy in the preceding six weeks.

Inclusion and exclusion criteria

The inclusion criteria were age ≥60 years, first episode of major depression after 60 years of age, the diagnosis confirmed by a consultant psychiatrist using the ICD-10 criteria, Mini-Mental State Examination (MMSE) score ≥24 (to exclude moderate to severe cognitive impairment), and consent to participate in the study and comply with follow-up assessments.

The exclusion criteria were a history of bipolar disorder, psychosis, substance dependence (except nicotine), or neurocognitive disorder; active inflammatory or autoimmune disease; recent history of acute infection or chronic inflammatory illness; use of anti-inflammatory or immunosuppressive drugs in the past four weeks; and comorbid severe medical illness that would interfere with study participation.

Sample size and enrollment

Using the formula n = Z²P(1−P)/d² (Z = 1.96 for 95% confidence, P = 10% expected prevalence, d = 0.01 precision), the minimum sample required was calculated as 36. To allow for attrition, 124 individuals were screened; 64 met the eligibility criteria and were enrolled in the cohort. Of these, 25 participants completed the full eight-week follow-up and were included in the final analysis [18].

Baseline assessment

At baseline, all participants underwent a standardized clinical and neuropsychiatric evaluation. Data collected included demographic variables, age at onset of depression, duration of illness, current and past medication history, medical comorbidities, and cerebrovascular risk quantified using the Framingham Stroke Risk Profile [19]. Depression severity was measured using the HAMD-17 [20]. Vital signs and relevant physical examination findings were recorded. Age at onset and illness duration were determined from patient reports and clinical records.

Exposure and outcome definition

The principal exposure of interest was baseline serum high-sensitivity CRP measured before the initiation of antidepressant therapy. The primary outcome was clinical antidepressant response at eight weeks, assessed by change in HAMD-17 score. Participants were classified as responders (e.g., ≥50% reduction in HAMD-17) or non-responders according to pre-specified response criteria. This longitudinal cohort design allowed assessment of whether baseline CRP predicted subsequent antidepressant response during the eight-week follow-up [21].

Intervention and follow-up

Following baseline assessment and blood sampling, all participants were initiated on escitalopram to ensure uniformity in the pharmacological intervention across the study cohort. The choice of escitalopram was based on its established efficacy, favorable tolerability profile, and common use in clinical practice for LOD. Dosing followed standard clinical guidelines, typically starting at 10 mg/day and adjusted (within the 10-20 mg/day range) according to individual clinical response and tolerability. Participants were managed as per routine clinical practice. The cohort was followed prospectively for eight weeks, and depression severity was reassessed at the end of follow-up using the HAMD-17 by the same trained evaluator to maintain measurement consistency.

CRP measurement

Blood samples for CRP were collected at the initial visit before starting antidepressant therapy. Participants avoided anti-inflammatory medications for at least 72 hours before sampling. Serum was separated (centrifugation at 3,000× g for 10 minutes) and stored at −20°C until analysis. High-sensitivity CRP was measured using latex-enhanced immunoturbidimetry (Infinite Turbilatex CRP, Accurex Biochemical Pvt. Ltd., Mumbai, India). A CRP value ≤5 mg/L was considered within the reference range.

Ethical approval

The study protocol was reviewed and approved by the Institutional Ethics and Scientific Review Boards of MGM Medical College, Indore (approval number: 42).

Statistical analysis

Data were analyzed using SPSS version 21.0 (IBM Corp., Armonk, NY, USA). Continuous variables are presented as mean ± SD and categorical variables as frequencies and percentages. Between-group comparisons (responders vs. non-responders) used Student’s t-test for normally distributed variables and the Mann-Whitney U test for non-normal data. Spearman’s rank correlation coefficient assessed the relationship between baseline serum CRP and HAMD-17 scores. A two-sided p-value <0.05 was considered statistically significant.

## Results

The baseline characteristics of the antidepressant responders and non-responders are shown in Table 1.

**Table 1:** Sample distribution according to sociodemographic variables. *: Independent t-test and chi-square test were applied for statistical analysis. A p-value <0.05 was considered statistically significant. NS: not significant

Variable	Non-responder (n = 19)	Responder (n = 6)	P-value (χ^2^/t)*
Age, years, mean (SD)	65.13 ± 6.14	63.51 ± 4.83	0.56 (NS)
Education, years, mean (SD)	3.41 ± 5.04	7.81 ± 4.93	0.07 (NS)
Sex, N (%)			0.42 (NS)
Male	8 (42%)	3 (50%)	
Female	11 (58%)	3 (50%)	
Marital status, N (%)			0.64 (NS)
Married	16 (84%)	5 (83%)	
Widowed	3 (16%)	1 (17%)	
Religion, N (%)			0.37 (NS)
Hindu	14 (74%)	4 (67%)	
Muslim	5 (26%)	2 (33%)	
Family type			0.57 (NS)
Nuclear	11 (58%)	3 (50%)	
Extended/Joint	8 (42%)	3 (50%)	
Residence, N (%)			0.37 (NS)
Urban	14 (74%)	4 (67%)	
Rural	5 (26%)	2 (33%)	

Sociodemographic profile

**Figure 1:**
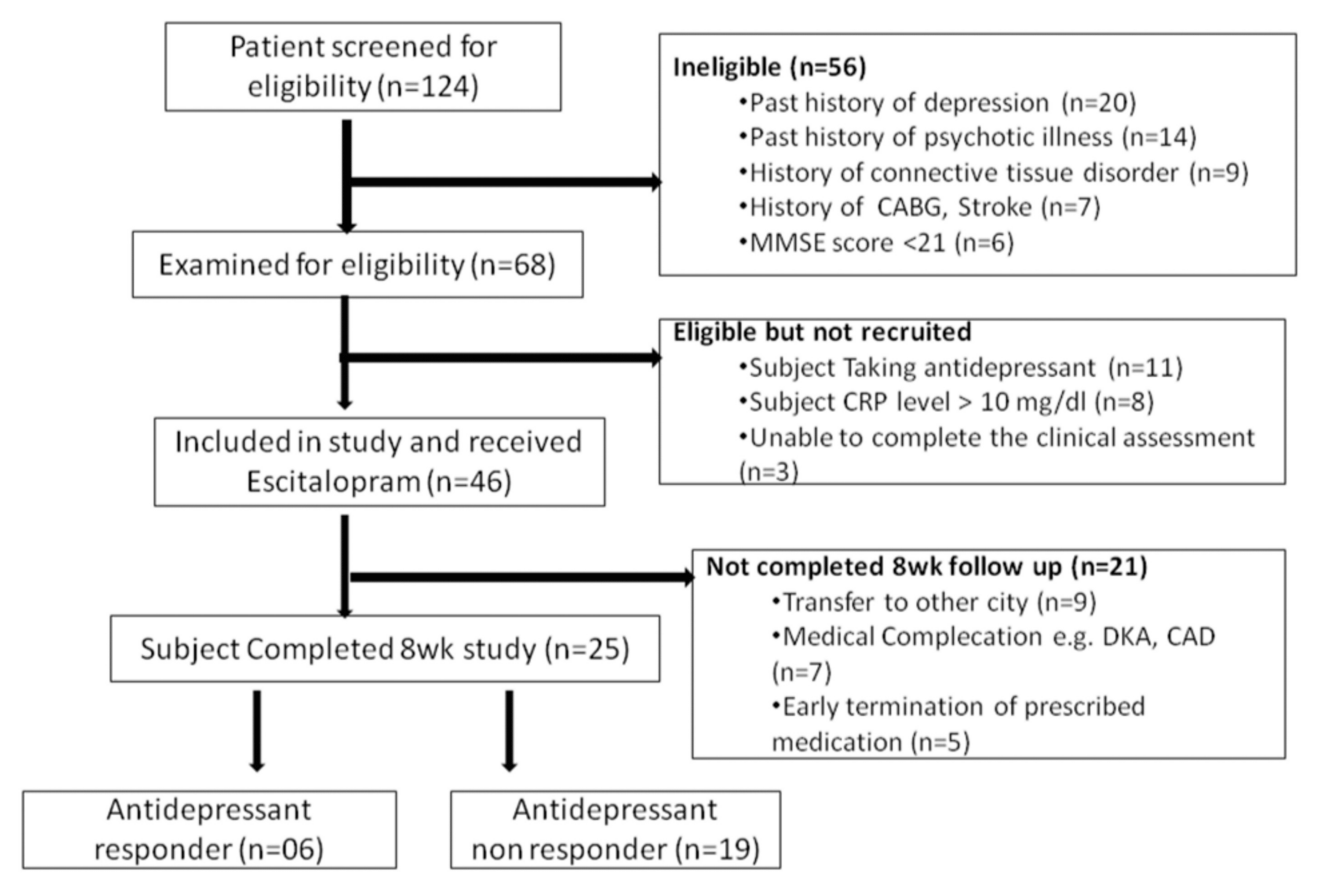
Flowchart of the participants included in study. CABG: coronary artery bypass grafting; MMSE: Mini-Mental State Examination; CRP: C-reactive protein; DKA: diabetic ketoacidosis; CAD: coronary artery disease

A total of 124 participants were enrolled in the study, of whom 64 fulfilled the eligibility criteria. In total, 25 participants completed eight weeks of the study, and the data from these participants were included in the final analysis (Figure 1). The mean age of the study sample was 64.7 ± 5.8 years (n = 25). The study sample had a statistically significant higher number of female subjects.

Clinical characteristics

Table 2 shows that the study sample’s baseline Hamilton depression severity rating score was 18 ± 3, which shows moderate severity of depression. Medical comorbidities were present in 60% of participants. No participants were using statins and anti-inflammatory drugs during study participation. Participants were assessed for stroke risk using the Framingham study score, and no statistically significant difference was found between antidepressant responders and non-responders.

**Table 2:** Clinical characteristics of the study cohort. *: Independent t-test and chi-square test were applied for statistical analysis. A p-value <0.05 was considered statistically significant. HAMD-17: 17-item Hamilton Rating Scale for Depression; NS: not significant

Variable	Non-responder (n = 19)	Responder (n = 6)	P-value (χ^2^/t)*
Age at onset, years, mean (SD)	64.24 ± 6.31	62.34 ± 4.91	0.51 (NS)
Duration of illness, month, mean (SD)	10.11 ± 8.13	9.70 ± 6.32	0.35 (NS)
Family history of psychiatric illness			0.64 (N.S.)
Absent	16 (84%)	5 (83%)	
Present	3 (16%)	1 (17%)	
Family history of substance use			0.12 (NS)
Absent	13 (68%)	6 (100%)	
Present	6 (32%)	0	
History of precipitating factors			0.41 (NS)
Absent	17 (89%)	6 (100%)	
Present	2 (11%)	0	
Medical diagnosis			0.70 (NS)
Absent	8 (42%)	2 (33%)	
Present	11 (58%)	4 (67%)	
Alcohol drinking			0.37 (NS)
Absent	18 (95%)	5 (83%)	
Present	1 (5%)	1 (17%)	
Recent hypertension diagnosis			0.96 (NS)
Absent	16 (84%)	5 (83%)	
Present	3 (16%)	1 (17%)	
Diabetes mellitus			0.94 (NS)
Absent	13 (68%)	4 (67%)	
Present	6 (32%)	2 (33%)	
Cigarette smoking			0.41 (NS)
Absent	17 (89%)	6 (100%)	
Present	2 (11%)	0	
Exercise level			0.37 (NS)
Very light	0	0	
Light	7 (37%)	1 (17%)	
Light plus	4 (21%)	3 (50%)	
Moderately vigorous	8 (42%)	2 (33%)	
Body mass index, kg/m^2^, mean (SD)	27.72 ± 3.93	26.42 ± 5.15	0.50 (NS)
Framingham study score, mean (SD)	6.17 ± 3.31	7.00 ± 3.01	0.74 (NS)
Mini-Mental Status Examination (MMSE)	24.84 ± 2.02	26.51 ± 2.78	0.11 (NS)
HAMD-17 score at baseline	18.64 ± 3.12	16 ± 1.9	0.06 (NS)
HAMD-17 score at 8 weeks	7.42 ± 1.21	8.33 ± 0.81	0.10 (NS)
CRP level, mg/dL, mean (SD)	6.27 ± 1.58	3.8 ± 1.4	0.002

Mean CRP level in the non-responder group was 6.27 ± 1.58 mg/dL, and in the responder group was 3.8 ± 1.4 mg/dL, which was statistically significant. Spearman’s rank correlation was computed to assess the relationship between CRP level and antidepressant responsiveness. There was a negative correlation between the two variables (r(df) = 0.588, p = 0.02).

## Discussion

Subjects with LOD typically had a longer duration of illness and moderate levels of depression severity [22]. Additionally, the majority of study participants in the antidepressant responder and non-responder groups had comorbid medical conditions. All participants were administered an eight-week course of antidepressant treatment. More than half of the study participants completed the study, while approximately 46% dropped out due to various reasons, such as transfer to another city, medical complications (e.g., coronary artery disease) during the study, and early termination of prescribed medication. The overall response rate to antidepressant treatment was 24%, which was significantly lower than the response rate in EOD, with the finding consistent with a previous study [23].

The study found that CRP levels were 60% higher in non-responders to antidepressant treatment than in responders, and there was a statistically significant (p < 0.05) difference in CRP levels between these two groups. Additionally, the baseline CRP level had a negative correlation with antidepressant responsiveness (r = -0.6, p < 0.05).

The potential explanation for the elevated CRP level is that subclinical vascular dysregulations, such as atherosclerosis, have been found to be associated with LOD [24,25]. This vascular damage could be a result of increased inflammation, as immune dysregulation is critically involved in vascular disease [14]. Furthermore, recent studies have suggested that variants of the CRP gene may influence circulating CRP levels and appear to be independent susceptibility factors for late-life depression [16]. Therefore, the elevated CRP level may serve as an independent marker of antidepressant response in late-onset depressive episodes.

The study has several strengths. First, it exclusively included patients with LOD, and their diagnosis was established based on diagnostic criteria rather than using depression rating scales. Second, the study participants were systematically recruited, and based on the data, the sample appears to be representative of an Indian population of patients at a tertiary care psychiatric hospital with a high prevalence of comorbid medical illnesses [26]. Third, the use of the inflammatory marker CRP is advantageous due to its easy availability. CRP can be obtained from a non-fasting peripheral blood sample, and high-sensitivity assays are widely available in most medical laboratories. Fourth, to adjust for vascular confounding factors in the case and control groups, the Framingham vascular risk factor scale was used. Finally, the MMSE score was used in addition to CRP to assess age-related dementia changes. The assessment was conducted in two stages: at baseline and eight weeks after the antidepressant response.

The study has a few limitations that should be taken into consideration. First, the sample size was small. Second, the interviewers were not blinded to the age of the participants, which might have influenced the results. Additionally, the study did not evaluate other potential factors such as stress, childhood adversities, social support, or positive life events that could be related to the age of onset. Another limitation is that the absence of other medical disorders was based on self-reported history rather than clinical workup. Finally, the study focused on patients treated at a tertiary psychiatric hospital, which limits the generalizability of the findings to patients with milder depression who receive treatment in primary care.

## Conclusions

LOD is associated with unique challenges due to higher medical comorbidities and reduced treatment responsiveness. CRP measurement offers a simple, cost-effective approach to predicting antidepressant response, enabling more tailored and effective management in this population.

## References

[1] (2022). Global, regional, and national burden of 12 mental disorders in 204 countries and territories, 1990-2019: a systematic analysis for the Global Burden of Disease Study 2019. Lancet Psychiatry.

[2] Barua A, Ghosh MK, Kar N, Basilio MA (2011). Prevalence of depressive disorders in the elderly. Ann Saudi Med.

[3] Panza F, Frisardi V, Capurso C (2010). Late-life depression, mild cognitive impairment, and dementia: possible continuum?. Am J Geriatr Psychiatry.

[4] Lebowitz BD, Pearson JL, Schneider LS (1997). Diagnosis and treatment of depression in late life. Consensus statement update. JAMA.

[5] Blazer DG, Hybels CF, Fillenbaum GG, Pieper CF (2005). Predictors of antidepressant use among older adults: have they changed over time?. Am J Psychiatry.

[6] Steinert C, Hofmann M, Kruse J, Leichsenring F (2014). The prospective long-term course of adult depression in general practice and the community. A systematic literature review. J Affect Disord.

[7] Reddy MS (2010). Depression: the disorder and the burden. Indian J Psychol Med.

[8] Whyte EM, Dew MA, Gildengers A, Lenze EJ, Bharucha A, Mulsant BH, Reynolds CF (2004). Time course of response to antidepressants in late-life major depression: therapeutic implications. Drugs Aging.

[9] Rush AJ, Fava M, Wisniewski SR (2004). Sequenced treatment alternatives to relieve depression (STAR*D): rationale and design. Control Clin Trials.

[10] Reynolds CF 3rd, Frank E, Kupfer DJ, Thase ME, Perel JM, Mazumdar S, Houck PR (1996). Treatment outcome in recurrent major depression: a post hoc comparison of elderly ("young old") and midlife patients. Am J Psychiatry.

[11] Burvill PW, Hall WD, Stampfer HG, Emmerson JP (1991). The prognosis of depression in old age. Br J Psychiatry.

[12] Alexopoulos GS, Chester JG (1992). Outcomes of geriatric depression. Clin Geriatr Med.

[13] Al-Harbi KS (2012). Treatment-resistant depression: therapeutic trends, challenges, and future directions. Patient Prefer Adherence.

[14] Alexopoulos GS, Bruce ML, Silbersweig D, Kalayam B, Stern E (1999). Vascular depression: a new view of late-onset depression. Dialogues Clin Neurosci.

[15] Su S, Miller AH, Snieder H (2009). Common genetic contributions to depressive symptoms and inflammatory markers in middle-aged men: the Twins Heart Study. Psychosom Med.

[16] Ancelin ML, Farré A, Carrière I, Ritchie K, Chaudieu I, Ryan J (2015). C-reactive protein gene variants: independent association with late-life depression and circulating protein levels. Transl Psychiatry.

[17] Rifai N, Tracy RP, Ridker PM (1999). Clinical efficacy of an automated high-sensitivity C-reactive protein assay. Clin Chem.

[18] Kohli C, Kishore J, Agarwal P, Singh SV (2013). Prevalence of unrecognised depression among outpatient department attendees of a rural hospital in Delhi, India. J Clin Diagn Res.

[19] Daniel WW (1999). Biostatistics: A Foundation for Analysis in the Health Sciences. https://books.google.co.in/books?hl=en&lr=&id=PON1DwAAQBAJ&oi=fnd&pg=PR7&dq=Daniel+WW,+editor.+7th+ed:+New+York:+John+Wiley+%26+Sons.+1999.&ots=a8_wd_mqMw&sig=QPxqEMGcifbOgT9yyeHj5Rh_eHo#v=onepage&q&f=false.

[20] HA M (1960). A rating scale for depression. J Neurol Neurosurg Psychiatry.

[21] Stassen HH, Delini-Stula A, Angst J (1993). Time course of improvement under antidepressant treatment: a survival-analytical approach. Eur Neuropsychopharmacol.

[22] Spijker J, de Graaf R, Bijl RV, Beekman AT, Ormel J, Nolen WA (2002). Duration of major depressive episodes in the general population: results from The Netherlands Mental Health Survey and Incidence Study (NEMESIS). Br J Psychiatry.

[23] Srivastava S, Kumar A, Khurana H (2015). Short-term course and outcome of late-life depression. J Geriatr Ment Health.

[24] Seldenrijk A, van Hout HP, van Marwijk HW (2011). Carotid atherosclerosis in depression and anxiety: associations for age of depression onset. World J Biol Psychiatry.

[25] Smith PJ, Blumenthal JA, Babyak MA (2009). Intima-media thickness and age of first depressive episode. Biol Psychol.

[26] Taylor WD, McQuoid DR, Krishnan KR (2004). Medical comorbidity in late-life depression. Int J Geriatr Psychiatry.

